# The C-terminal domain of *Clostridium perfringens* alpha toxin as a vaccine candidate against bovine necrohemorrhagic enteritis

**DOI:** 10.1186/s13567-016-0336-y

**Published:** 2016-04-27

**Authors:** Evy Goossens, Stefanie Verherstraeten, Bonnie R. Valgaeren, Bart Pardon, Leen Timbermont, Stijn Schauvliege, Diego Rodrigo-Mocholí, Freddy Haesebrouck, Richard Ducatelle, Piet R. Deprez, Filip Van Immerseel

**Affiliations:** Department of Pathology, Bacteriology and Avian Diseases, Faculty of Veterinary Medicine, Ghent University, Salisburylaan 133, B-9820 Merelbeke, Belgium; Department of Internal Medicine and Clinical Biology of Large Animals, Faculty of Veterinary Medicine, Ghent University, Salisburylaan 133, B-9820 Merelbeke, Belgium; Department of Surgery and Anesthesia of Domestic Animals, Faculty of Veterinary Medicine, Ghent University, Salisburylaan 133, B-9820 Merelbeke, Belgium

## Abstract

Bovine necrohemorrhagic enteritis is caused by *Clostridium perfringens* and leads to sudden death. Alpha toxin, together with perfringolysin O, has been identified as the principal toxin involved in the pathogenesis. We assessed the potential of alpha toxin as a vaccine antigen. Using an intestinal loop model in calves, we investigated the protection afforded by antisera raised against native alpha toxin or its non-toxic C-terminal fragment against *C. perfringens*-induced intestinal necrosis. Immunization of calves with either of the vaccine preparations induced a strong antibody response. The resulting antisera were able to neutralize the alpha toxin activity and the *C. perfringens*-induced endothelial cytotoxicity in vitro. The antisera raised against the native toxin had a stronger neutralizing activity than those against the C-terminal fragment. However, antibodies against alpha toxin alone were not sufficient to completely neutralize the *C. perfringens*-induced necrosis in the intestinal loop model. The development of a multivalent vaccine combining the C-terminal fragment of alpha toxin with other *C. perfringens* virulence factors might be necessary for complete protection against bovine necrohemorrhagic enteritis.

## Introduction

*Clostridium perfringens* is a Gram-positive, spore-forming, anaerobic bacterium. It is a normal component of the intestinal microbiota of animals, including humans. It secretes several toxins and enzymes that cause different forms of tissue damage [1–3]. Consequently, it can cause a variety of diseases in various vertebrates [2]. The differences in virulence properties between *C.perfringens* isolates are largely due to differences in toxin production. Alpha toxin and perfringolysin O have been identified as the principal toxins involved in gas gangrene caused by *C. perfringens* as well as in bovine necrohemorrhagic enteritis [4]. Gas gangrene is a frequently lethal histotoxic infection of humans and animals characterized by rapid tissue destruction and impaired immune response [5, 6]. Bovine necrohemorrhagic enteritis (bovine enterotoxaemia) is an enteric disease of veal calves and beef type suckling calves and is characterized by hemorrhagic to necrotizing enteritis. Calves often die without premonitory signs [4, 7–9].

We recently showed that vaccination of calves with a mixture of native toxins from *C.perfringens* induces antibodies that protect against *C. perfringens* challenge in an intestinal loop model of bovine necrohemorrhagic enteritis (Goossens et al., provisionally accepted). Although both alpha toxin and perfringolysin O are involved in the pathogenesis of gas gangrene, immunization against alpha toxin alone provides good protection against experimental gas gangrene [6, 10, 11]. Moreover, Evans showed that antiserum raised against alpha toxin was highly effective in protecting guinea pigs against experimental gas gangrene, whereas antiserum to perfringolysin O was not protective against *C. perfringens* type A infection, and it did not enhance the protective action of alpha toxin antiserum [12]. Studies on gas gangrene cannot be directly extrapolated to bovine necrohemorrhagic enteritis, but these findings indicate that alpha toxin vaccines could provide protection against diseases in which alpha toxin is critically important.

Here, we tested vaccine preparations based on alpha toxin, the major toxin produced by *C.perfringens* type A. Since native toxins are not safe, we used the enzymatically inactive C–terminal domain of alpha toxin (Cpa_247–370_). This component is non-toxic and has been shown to provide protection against *C.perfringens* type A gas gangrene in a mouse model, and it is known to elicit protective immunity against a broad range of clostridial phospholipase C toxins [10, 13, 14]. In addition, mice vaccinated with Cpa_247–370_ were protected against challenge with alpha toxin derived from a calf necrohemorrhagic enteritis isolate [15].

The aim of this study was to evaluate whether the non-toxic C-terminal fragment of alpha toxin could be a candidate for effective vaccination of calves against bovine necrohemorrhagic enteritis.

## Materials and methods

All experimental protocols were approved by the ethics committee of the Faculty of Veterinary Medicine, Ghent University (EC2011/024, EC2012/056, EC2013/38, EC2013/39 and EC2013/187). All animal experiments were carried out in accordance with the approved guidelines.

### Bacterial strains

The *C. perfringens* strains were wild-type strain JIR325, the *plc* mutant JIR4107 (∆*plc*), and the *C. perfringens* JIR4107 derivatives carrying either the *plc*^+^ plasmid (complemented strain JIR4121) or the empty shuttle vector (complementation control JIR4120) (Table 1) [16, 17]. The strains were cultured anaerobically at 37 °C in Brain Heart Infusion broth (BHI, Oxoid, Basingstoke, UK) containing 0.375% glucose. To culture JIR4120 [∆*plc*; (shuttle vector)] and JIR4121 (complemented strain), the medium was supplemented with chloramphenicol (30 µg/mL). The logarithmic phase cultures used in intestinal loop experiments did not contain antibiotics. To determine the alpha toxin concentration in the culture supernatant, cell-free supernatants were obtained by centrifugation followed by filtration of the supernatants through a 0.22-µm filter. The alpha toxin concentration in the bacterial supernatants was measured using the Bio-X α-toxin ELISA kit (Bio-X Diagnostics, Jemelle, Belgium) and twofold serial dilutions of the alpha toxin standard (220 × 10^−3^‒0.8 × 10^−3^ U/mL of phospholipase C type I; Sigma-Aldrich, St Louis, MO, USA) as previously described [18].

**Table 1:** ***Clostridium perfringens*** **strains used in the study**

Strain^**a**^	Strain number	Phenotype	Origin	Toxin genes	Alpha toxin (*10^−3^ U/mL) mean ± SEM	Ref.
Wild-type	JIR325	Wild-type	Strain 13^b^	*plc*	31.392 ± 0.079	[16]
∆*plc*	JIR4107	Alpha toxin-deficient	JIR325 Δ*plc*		<0.8	[17]
∆*plc* (shuttle vector)	JIR4120	Alpha toxin-deficient with shuttle vector	JIR4107(pJIR418)		<0.8	[17]
complemented	JIR4121	Alpha toxin- complemented	JIR4107(pJIR443)	*plc*	28.32 ± 0.38	[17]

^a^ *plc*: alpha toxin gene.

^b^JIR325: a rifampicin and nalidixic acid-resistant derivative of strain 13, a *C. perfringens* strain originally isolated from soil.

### The role of *C. perfringens* alpha toxin in the induction of necrotic lesions in an intestinal loop model

To confirm the role of alpha toxin in the induction of necrotic lesions in an intestinal loop model, seven intestinal loop experiments were conducted using the wild-type *C.perfringens* strain JIR325 and the alpha toxin-deficient strain *C.perfringens* JIR4107. In two of the experiments, the *C. perfringens* JIR4107 derivatives carrying the empty shuttle vector (JIR4120) or the *plc*^+^ plasmid (JIR4121) were also included. The number of loops injected with each strain is shown in Table 2. In each calf, an equal number of control loops were injected with sterile bacterial growth medium supplemented with milk replacer. The experiments were performed according to a published protocol using seven healthy male Holstein–Friesian veal calves aged 3‒5 months [19]. Briefly, the calves were anesthetized and the small intestine was exteriorized. The loops were ligated and injected with logarithmic phase cultures combined with 25% commercial milk replacer (Vitaspray, Nuscience Drongen, Belgium) in sterile 0.9% NaCl solution, as described [19]. The animals were kept under anesthesia for 5 hours after inoculation, after which they were euthanized and samples were taken. Intestinal loop tissue samples were submerged in 4% (w/v) phosphate buffered formaldehyde. After fixation for 24 h, the samples were processed routinely, embedded in paraffin wax, sectioned, and stained with hematoxylin and eosin. Sections were evaluated in a blinded manner by a board certified pathologist for the presence of tissue necrosis (0 = absence of necrosis, 1 = necrotic lesions present).

**Table 2:** **The number of loops inoculated with each strain in the intestinal loop experiments to evaluate the role of** ***C. perfringens*** **alpha toxin in the induction of necrotic lesions**

Calf	Replicate loops/strain^a^				
	JIR325	JIR4107	JIR4120	JIR4121	BHI
1	3	3	/	/	3
2	3	3	/	/	3
3	3	3	/	/	3
4	5	5	/	/	5
5	5	5	/	/	5
6	5	5	5	5	5
7	5	5	5	5	5

Seven intestinal loop experiments were conducted. The number of intestinal loops that were injected per animal are shown.

^a^JIR325: wild-type *C. perfringens*; JIR4107: alpha toxin-deficient; JIR4120: alpha toxin-deficient strain carrying the empty shuttle vector; JIR4121: alpha toxin-complemented strain; BHI: sterile bacterial growth medium.

### Preparation of recombinant alpha toxin

Alpha toxin was expressed in *Escherichia coli* using the pBAD TOPO^®^ TA Expression Kit (Invitrogen, Paisley, UK). A fragment encoding the *C. perfringens* alpha toxin (*plc* gene; GenBank accession number BAB79742) was amplified from the DNA of *C. perfringens* JIR325 by PCR using a DNA polymerase with proofreading activity (Accuzyme, Bioline, Randolph, MA, USA). The forward primer (5′- G **TGA** GAG GAG GAT ATA AAA **ATG** AAA AGA AAG ATT TGT AAG GCG -3′) contained an in-frame stop codon and translation re-initiation sequence to remove the N-terminal leader and allow native protein expression. The reverse primer (5′- G TTT CTT TTT TAT ATT ATA AGT TGA ATT TCC TGA AAT CCA CTC -3′) excluded the native *plc* gene stop codon and included the C-terminal V5 epitope and polyhistidine region for affinity purification. The resulting PCR product was incubated with *Taq* polymerase for 10 min at 72 °C (5 U; Promega, Madison, WI, USA) to add 3′ A-overhangs, cloned into the pBAD-TOPO expression vector, and transformed into One Shot TOP10^®^*E. coli.* The correct orientation of the alpha toxin insert was verified by Sanger sequencing.

*Escherichia coli* carrying the pBAD-alpha toxin vector was grown at 37 °C to an OD_600_ of 0.4‒0.5 in Terrific Broth supplemented with 100 µg/mL ampicillin. Expression of recombinant *C. perfringens* alpha toxin was induced for 4 h by adding L-arabinose to a final concentration of 0.002% (w/v). Bacteria were harvested by centrifugation and lysed enzymatically using BugBuster (Invitrogen). Alpha toxin was purified on a Ni-Sepharose column (His Gravitrap, GE Healthcare Bio-Sciences AB, Uppsala, Sweden) according to the manufacturer’s instructions. Subsequently, the protein was dialyzed against PBS, purity was analyzed using SDS-PAGE, and protein concentration was measured using BCA protein assay (Thermo Fisher Scientific, Waltham, MA, USA).

### Vaccine preparation and immunization

The recombinant carboxy-terminal domain of alpha toxin fused to glutathione-S-transferase (GST) was kindly provided by Prof. Titball, University of Exeter, UK. This Cpa_247–370_ was produced in *E. coli* and was therefore devoid of any other *C. perfringens* proteins [20]. Recombinant native alpha toxin (rCpa) and Cpa_247–370_ were formulated with the adjuvant QuilA (Brenntag Biosector, Frederikssund, Denmark) in PBS. Each animal was injected with 1.5 mL of the filter-sterilized (0.2 µm) formulation containing 350 µg antigen and 750 µg QuilA. Control animals received 750 µg QuilA in 1.5 mL PBS.

Six male Holstein–Friesian calves aged 2 months were used. They were housed on straw and received water and hay *at libitum*, and concentrates adjusted to the body weight.

For each antigen (rCpa, Cpa_247–370_ or QuilA control), two calves were immunized subcutaneously in the neck. The calves received a primer vaccination at the age of 2 months, and booster immunizations 14 and 28 days later.

### Enzyme-linked immunosorbent assay

The immune response following vaccination was measured using serum samples obtained 2 weeks after the final booster immunization. Alpha toxin-specific antibody levels were determined by the end-point dilution method using a blocking ELISA (*C. perfringens* alpha toxin serological ELISA kit, Bio-X Diagnostics). Sera were used at a dilution 1:50 and assays were performed in duplicate. The specific antibody level was expressed as percent inhibition according to the following formula:  % inhibition = [(OD negative − OD sample)/OD negative] × 100.

### Neutralization of the hemolytic activity of wild-type *C. perfringens* JIR325 alpha toxin on blood agar plates in vitro

Incubation of cell-free supernatants of the wild-type strain JIR325 (concentrated tenfold using Vivaspin, Sartorius Stedim Biotech GmbH, Göttingen, Germany) on sheep blood agar at 37 °C overnight results in an inner, complete zone of hemolysis caused by perfringolysin O and a less complete outer zone caused by alpha toxin. The sera’s ability to neutralize alpha toxin activity was assessed by incubating the JIR325 supernatant with an equal volume of the pooled sera from the two animals that were vaccinated with either a given vaccine or the adjuvant QuilA for 30 min at 37 °C. Ten-microliter drops of these mixtures were spotted on sheep blood agar and hemolysis was assessed after overnight incubation. The test was performed in triplicate using supernatants of *C. perfringens* JIR325 from three independent biological replicates.

### Neutralization of alpha toxin activity on egg yolk lipoproteins

Alpha toxin activity was determined in duplicate in a 96-well microtiter plate by evaluating its effect on egg yolk lipoproteins as previously described [21]. The neutralizing ability of sera was assessed by pre-incubating a twofold dilution series of the sera (two wells per dilution) with a constant amount of alpha toxin (10 µg/mL in PBS) for 30 min at 37 °C before adding 10% egg yolk emulsion. To prepare the yolk emulsion, fresh egg yolk was centrifuged (10 000 × *g* for 20 min at 4 °C) and diluted 1:10 in PBS. After incubation of the 96-well plates at 37 °C for 1 h, absorbance was measured at 650 nm. Alpha toxin activity was indicated by the development of turbidity, which increases absorbance. The inhibitory capacity of the antiserum was expressed as the serum dilution that inhibited 50% of the alpha toxin activity. This was determined by applying a Hill function to the concentration–response data (GraphPad Prism 5, GraphPad Software, San Diego, CA, USA). The test was performed in duplicate.

### Neutralization of *C. perfringens* cytotoxicity to bovine endothelial cells

Primary bovine umbilical vein endothelial cells (BUVEC) were isolated from umbilical cord veins by an adapted procedure [4] based on the method of Jaffe et al. [22]. The toxicity of *C. perfringens* supernatant to cultured bovine endothelial cells has been reported [4]. The ability of the antisera to neutralize the *C. perfringens* cytotoxicity to BUVECs was determined using a Neutral Red Uptake assay (NRU) [23]. Briefly, BUVEC cells were seeded in 96-well tissue culture plates at a density of 10^5^ cells per well and cultured for 24 h to obtain cells in the exponential growth phase. The neutralizing ability of the sera was assessed by pre-incubating a twofold dilution series of the sera (100–0.4%) prepared in serum-free cell culture medium with an equal volume of undiluted *C. perfringens* supernatant. After 30 min at 37 °C, the cells were treated for 2 h with 100 µL of the supernatant‒serum mixture, followed by a standard NRU assay. The inhibitory capacity of the antiserum was expressed as the last dilution associated with 100% cell viability. As a positive control, cells were treated with *C. perfringens* supernatant pre-incubated with serum-free medium. Untreated cells incubated with serum-free medium served as a negative control. The test was performed in duplicate.

### Neutralization of necrotic lesion development in the intestinal loop model

To study the protection against *C. perfringens*-induced necrosis provided by the antisera obtained from calves vaccinated with the respective vaccines, three intestinal loop experiments were performed using three male Holstein–Friesian calves aged 3 months. In each of the three intestinal loop experiments, the sera for each vaccine were pooled. Intestinal loops were inoculated with a wild-type strain (JIR325) combined with 25% commercial milk replacer suspended in sterile NaCl solution. Before inoculation, serum from calves immunized with the different vaccine preparations was added to the NaCl solution containing milk replacer to a final concentration of 6% serum (v/v). In each calf, five intestinal loops were injected with anti-Quil A, five with anti-native alpha toxin, and five with anti-C-terminal fragment of alpha toxin. Moreover, five control loops per calf were injected with *C. perfringens* without addition of serum (positive control) and five with sterile bacterial growth medium (negative control). This totaled 25 injected loops per calf. Samples were collected and scored as described for the intestinal loop experiments using the alpha toxin-deficient strain.

### Statistical analysis

Differences in the development of necrotic loops between the wild-type and the mutant *C.perfringens* strains were analyzed using multivariable logistic regression. The protective effect of the different antisera against development of intestinal necrosis in the loop model was determined by multivariable logistic regression. To account for clustering of loops within a calf, a fixed factor was included describing in which calves the experiments were performed. Significance was set at *p* < 0.05 and analyses were performed in SPSS v. 22.0 (IBM Corporation, New York, USA). Results were reported as means and standard errors of the means (SEM).

## Results

### *Clostridium perfringens* alpha toxin-deficient strain has a decreased ability to cause necrotic lesions in an intestinal loop model

**Figure 1:**
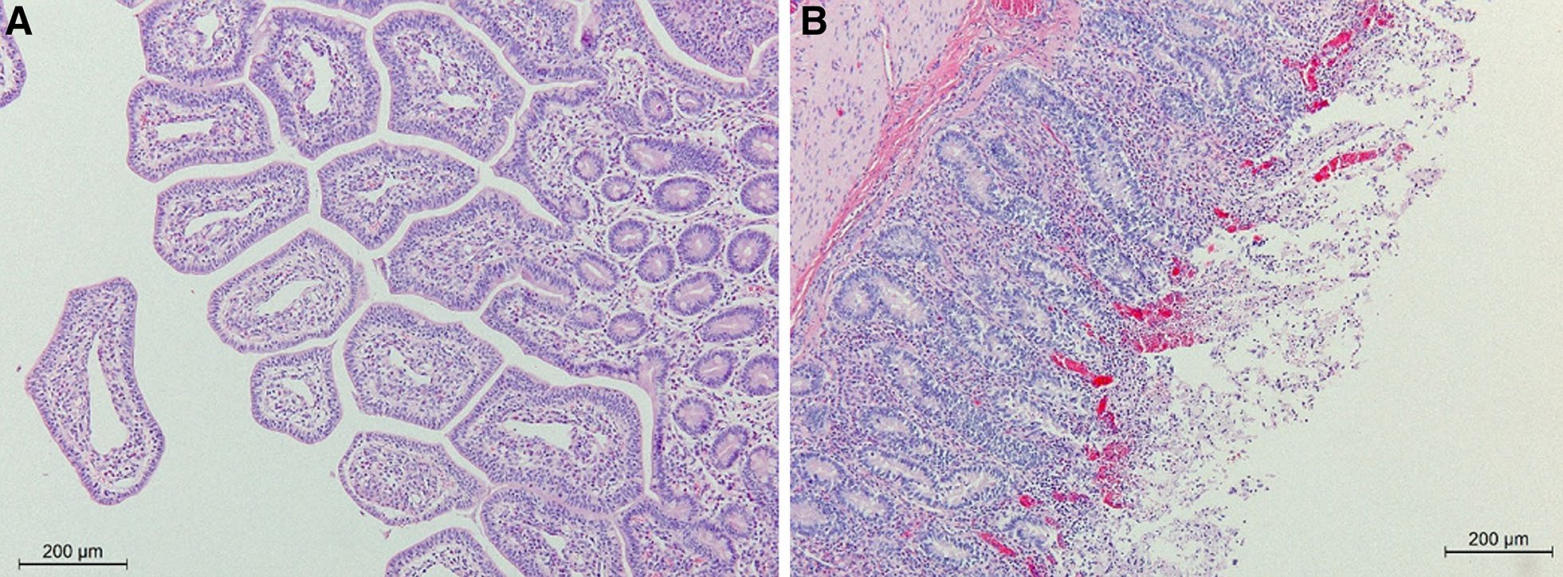
***C. perfringens*** **-induced necrosis in experimentally infected intestinal loops in calves.** (**A**) Representative histological section from an intestinal loop injected with sterile bacterial growth medium. There are no lesions in this negative control loop. (**B**) Representative histological section from an intestinal loop injected with the wild-type *C. perfringens* strain, showing hemorrhages and necrosis of the villi. HE, bars 200 µm.

**Figure 2:**
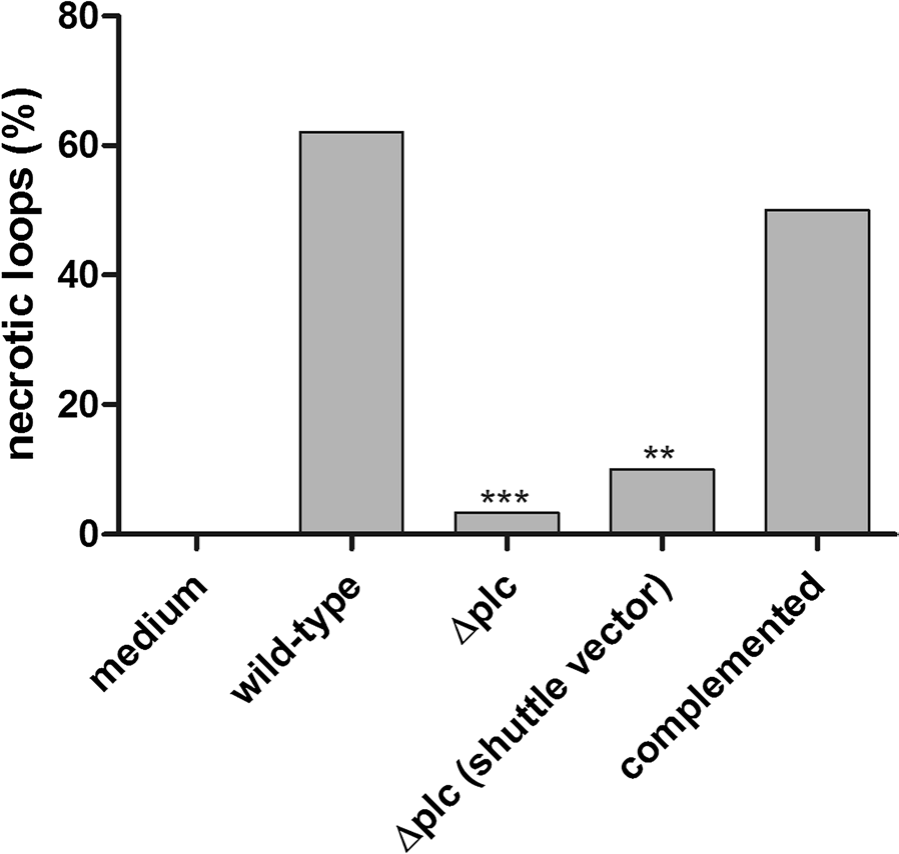
**Percentage of necrotic loops after** ***C. perfringens*** **inoculation.** Intestinal loops inoculated with sterile cell culture medium (*n* = 29), the wild-type strain (*n* = 29), the alpha toxin-deficient strain (Δ*plc*) (*n* = 29), the alpha toxin deficient strain carrying the empty shuttle vector [Δ*plc* (shuttle vector)] (*n* = 10) and the alpha toxin-complemented strain (*n* = 10) were histologically examined for the presence of tissue necrosis. The graph represents the percentage of loops in which necrotic lesions were present after 5 h of incubation with logarithmic stage cultures. ** 0.001 ≤ *P* < 0.01 and ***P < 0.001 indicate a significant difference relative to the loops inoculated with the wild-type strain.

A wild-type strain and an alpha toxin-deficient strain (∆*plc*) were tested in an intestinal loop model. The wild-type strain caused necrotic lesions in 62.1% (18/29) of the injected loops, whereas the alpha toxin-deficient strain induced necrosis in significantly fewer loops (3.4%; 1/29) (*p* < 0.001). To confirm the role of alpha toxin in lesion development by complementing the deficiency, the ∆*plc* derivatives carrying the empty shuttle vector (JIR4120) or the *plc*^+^ plasmid (JIR4121) were used. Necrotic lesions were observed in only one of the ten (10%) loops injected with the alpha toxin-deficient strain carrying the empty shuttle vector. This is significantly fewer than in the loops inoculated with the wild-type strain (62.1%; *p* = 0.008). The *plc*-complemented strain induced necrotic lesions in 50% (5/10) of the loops, which is comparable to the effect of the wild-type strain. No lesions were detected in the control loops treated with sterile bacterial culture medium (Figures 1 and 2).

### Antibody responses against alpha toxin in calves

After vaccination with native alpha toxin, the non-toxic C-terminal domain of alpha toxin or the adjuvant QuilA, serum antibodies produced against native alpha toxin were analyzed by ELISA. In all calves vaccinated with the native toxin or with the C-terminal domain, a strong antibody response against alpha toxin was detected 6 weeks after the first immunization. The calves vaccinated with the native toxin had antibody titers of 69.7 ± 7.8. Calves vaccinated with the non-toxic C-terminal domain of alpha toxin had antibody titers of 91.1 ± 1.6. No anti-alpha toxin response was measured in the calves vaccinated with the adjuvant QuilA.

### Neutralization of alpha toxin activity in vitro

**Figure 3:**
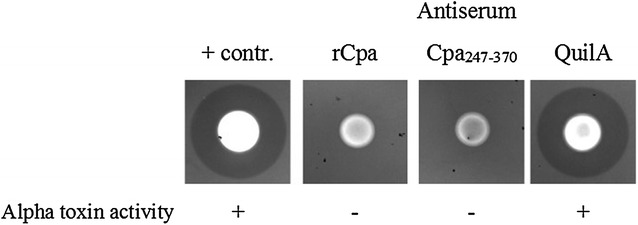
**In vitro** **neutralization of the hemolytic activity of the alpha toxin of** ***C.*** ***perfringens.*** Supernatants of *C. perfringens* (+contr.) were either left untreated or were pre-incubated with serum from calves immunized with native alpha toxin (rCpa), the non-toxic C-terminal fragment of alpha toxin (Cpa_247–370_) or the adjuvant QuilA. They were then spotted on sheep blood agar and incubated overnight at 37 °C. Neutralization of alpha toxin activity results in absence of an outer zone of hemolysis. +, no neutralization of toxin activity; −, complete neutralization of toxin activity. Representative pictures of one out of three independent experiments.

Sheep blood agar was used to examine in vitro neutralization of alpha toxin activity of a wild-type *C.perfringens* strain by sera from calves immunized with the native alpha toxin (rCpa) or the non-toxic C-terminal fragment of the alpha toxin (Cpa_247–370_). Plates treated with *C. perfringens* supernatant exhibited both the inner (perfringolysin O) and outer (alpha toxin) zones of hemolysis. Incubation of the supernatant with sera against either rCpa or Cpa_247–370_ did not result in an outer zone of hemolysis, indicating neutralization of alpha toxin activity. These sera had no effect on perfringolysin O activity. Incubation with sera from the control calves (QuilA) had no effect on *C. perfringens* toxin activities (Figure 3).

To determine whether the antisera against the vaccines can neutralize the lecithinase activity of alpha toxin, serial dilutions of the antisera were incubated with alpha toxin and its activity was measured on egg yolk lipoproteins. The sera of calves immunized with either the native alpha toxin (rCpa) or the C-terminal fragment of alpha toxin (Cpa_247–370_) decreased the activity of alpha toxin, with an inhibitory capacity of respectively 1189.0 ± 390.4 for anti-rCpa or 323.8 ± 133.3 for the sera raised against Cpa_247–370_. No effect on alpha toxin activity was observed after incubation with sera from calves immunized only with QuilA.

### Neutralization of the cytotoxicity of *C. perfringens* to bovine endothelial cells by anti-alpha toxin antisera

To determine whether the antisera against the vaccines can inhibit the cytotoxicity of *C. perfringens*, serial dilutions of the antisera were incubated with *C. perfringens* supernatant. Exposure of the endothelial cells to untreated supernatant resulted in 100% cell death. Antisera raised against either the native alpha toxin (rCpa) or the C-terminal fragment of alpha toxin (Cpa_247–370_) protected the endothelial cells from *C. perfringens* cytotoxicity. Sera from the control calves did not neutralize the *C. perfringens*-induced cytotoxicity. Pre-incubating the *C. perfringens* supernatant with a 288-fold dilution (±96) of the native alpha toxin antiserum resulted in 100% neutralization of cytotoxicity, whereas a 32-fold dilution (±0.0) of the antiserum against the C-terminal fragment (Cpa_247–370_) was needed to neutralize the cytotoxicity.

### Protective effect of anti-alpha toxin antisera against *C. perfringens*-induced necrosis in an intestinal loop model

**Figure 4:**
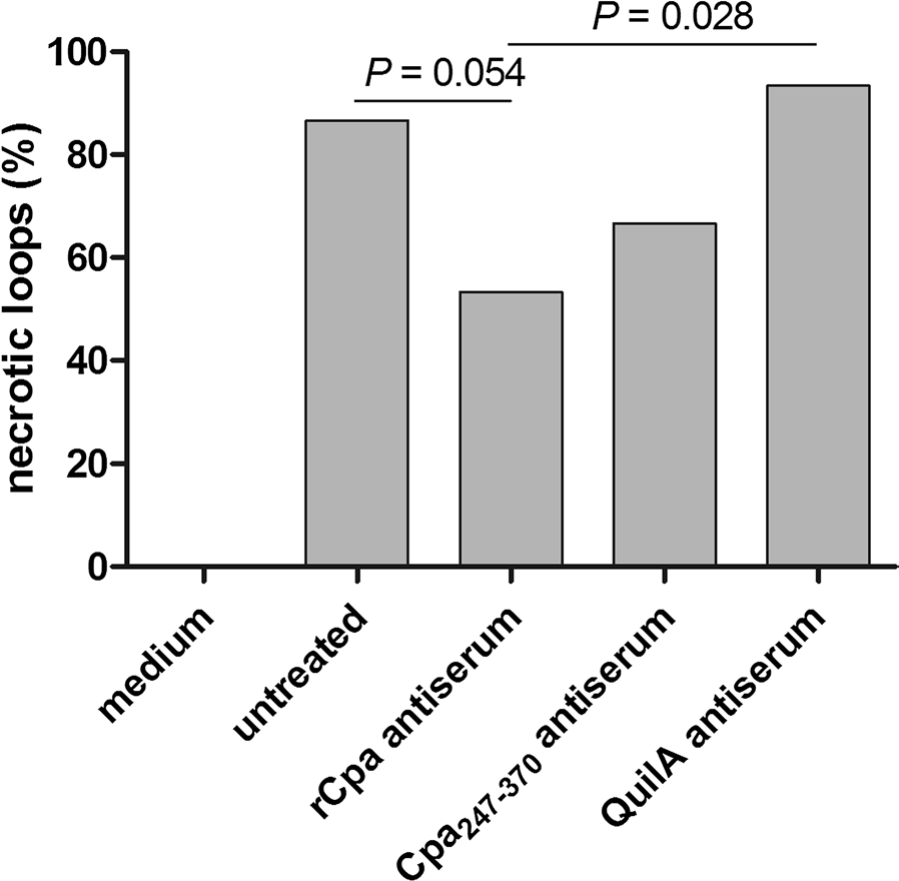
**Neutralization of the lesion-inducing potential of** ***C. perfringens.*** The graph represents the percentage of intestinal loops in which necrotic lesions were present after 5 h of incubation with five treatments: sterile culture medium, *C. perfringens* alone (untreated) or *C. perfringens* in combination with 6% antiserum to either native alpha toxin (rCpa antiserum), antiserum to the non-toxic C-terminal fragment of alpha toxin (Cpa_247–370_ antiserum), or antiserum from calves immunized with adjuvant only (QuilA antiserum). The graph represents the data from three intestinal loop experiments (total of 15 loops per condition).

Neutralization of the lesion-inducing potential of *C. perfringens* by sera raised against the respective vaccines was evaluated in the intestinal loop model. Thirteen of the fifteen (86.7%) positive control loops inoculated with *C. perfringens* developed necrosis. Injection of loops with *C. perfringens* combined with sera from control calves (immunized with the adjuvant QuilA) also resulted in a high percentage of necrotic loops (93.3% of the loops, 14/15). Injection of loops with *C. perfringens* combined with antisera raised against native alpha toxin (rCpa) resulted in significantly fewer necrotic loops as compared to the loops containing *C. perfringens* and the QuilA antisera (*p* = 0.028) and borderline significantly fewer necrotic loops as compared to the untreated loops (*p* = 0.054) (53.3% of the loops or 8/15). Antisera raised against the non-toxic C-terminal fragment of alpha toxin (Cpa_247–370_) did not significantly neutralize the lesion-inducing ability of *C. perfringens* (10/15 or 66.7% necrotic loops) (Figure 4).

## Discussion

Alpha toxin is involved in the induction of necrotic lesions in a calf intestinal loop model and is thus an important toxin in the pathogenesis of enterotoxaemia. We previously showed that alpha toxin production by *C. perfringens* is required for intestinal virulence by using a double-mutant *C. perfringens* strain devoid of alpha toxin and perfringolysin O, which was complemented for perfringolysin O to generate an alpha toxin-deficient phenotype [4]. In the present study, we supported our earlier conclusions by using an alpha toxin-mutant strain. Mutant strains are frequently used to evaluate the virulence effect of *C.perfringens* genes. A mutant strain was used to show that NetB is crucial for the induction of avian necrotic enteritis [24]. This approach also identified beta toxin as an essential virulence factor of *C. perfringens* type C in infected rabbits [25]. Moreover, Awad et al. used mutant strains to demonstrate that both alpha toxin and perfringolysin O are involved in the pathogenesis of gas gangrene [17, 26]. In our study, we confirmed that alpha toxin is required for intestinal virulence in a calf intestinal loop model. This conclusion was based on genetic evidence showing that an alpha toxin-deficient strain has a decreased ability to cause necrotic lesions in this model. The alpha toxin-complemented strain regained the ability to cause the disease, unambiguously fulfilling Falkow’s molecular Koch’s postulates [27].

In the present study, alpha toxin appeared to be a promising vaccine component against bovine necrohemorrhagic enteritis. Antisera raised against native alpha toxin reduced the lesion-inducing potential of *C. perfringens* in the intestinal loop model. However, alpha toxin is a potent dermonecrotic toxin that is not safe for use in calves. Alpha toxin can be rendered safe by formaldehyde treatment, but a well-known problem of this treatment is that it might reduce immunogenicity [5, 28–30]. Therefore, a recombinant *C.perfringens* alpha toxoid may be preferable to a formaldehyde toxoid. The immunogenicity of the C-terminal fragment of alpha toxin in calves was recently reported for the first time [31]. However, the ability of the antiserum derived after vaccination of calves with Cpa_247–370_ to neutralize the toxicity of *C. perfringens* to bovine cells or bovine intestine has not been evaluated [31]. Here, we report that the non-toxic C-terminal domain of alpha toxin (Cpa_247–370_) may be an effective alternative to the use of native alpha toxin. Indeed, calves immunized with the native alpha toxin or with the C-terminal domain of alpha toxin developed a strong immune response against alpha toxin. Nevertheless, compared to antisera against the native alpha toxin, sera from calves immunized with the C-terminal fragment of alpha toxin showed weaker inhibition of the alpha toxin activity and weaker neutralization of the *C. perfringens*-induced endothelial cytotoxicity in vitro. Additionally, the lesion-inducing potential of *C. perfringens* in the intestinal loop model was significantly reduced only by co-administration of antisera from animals vaccinated with the native alpha toxin.

The diminished protection afforded by antisera against the C-terminal domain may be attributed to the GST tag fused to the C-terminal domain of alpha toxin for protein purification purposes. Distortion of the conformation of the alpha toxin fragment by the GST tag has already been suggested in a previous study reporting that the untagged fragment was more protective against experimental gas gangrene than the C-terminal fragment fused to the GST tag [13]. In contrast to the C-terminal fragment of alpha toxin, the recombinant native alpha toxin used in this study was fused to a HIS tag for purification. This HIS tag is substantially smaller than the GST tag and is less likely to influence the conformation of the alpha toxin. This construct might generate more antibodies against the conformational epitopes that are important for protection. Alternatively, it may be that, in addition to antibodies directed to the C-terminal fragment of alpha toxin, also antibodies against the N-terminal fragment are needed to provide protection. However, a previous study showed that immunization with the N-terminal domain of alpha toxin was not protective against experimental gas gangrene [13]. It is believed that membrane binding induces a conformational change in the N-terminal domain from the closed to open configuration, which could reduce the affinity of antibodies raised against the N-terminal domain and complicates the development of protective antibodies against this N-terminal region [32, 33]. Moreover, the combination of both toxin domains as vaccine antigen is not straightforward because combination of both non-toxic fragments restores the biological activity of alpha toxin [34].

Total protection was not obtained even after vaccination with native alpha toxin. It is possible that not all alpha toxin was neutralized by the antisera, leaving residual active alpha toxin to exert cytotoxicity. We also do not know whether in the field serum antibodies leaking into the intestinal lumen after intestinal damage will be sufficient to inhibit alpha toxin and the induction of necrotic lesions. This should be tested in a subsequent study by performing intestinal loop experiments in immunized animals without adding antiserum to the ligated intestinal loops. It is possible that total protection against development of intestinal lesions also requires other neutralizing antibodies, for example, against perfringolysin O and/or other *C. perfringens* proteins. Therefore, other *C. perfringens* proteins in addition to alpha toxin and perfringolysin O might have to be incorporated in a vaccine to obtain complete protection. This is also the case for avian necrotic enteritis, where NetB is essential to cause disease, but vaccination with NetB provides only partial protection against *C. perfringens* challenge [35–37].

Endothelial damage is probably a key event in the pathogenesis of bovine necrohemorrhagic enteritis [4, 19]. Initial epithelial damage could enable alpha toxin to penetrate the epithelial barrier and to act on endothelial cells. In addition to other infectious agents, such as coccidia, enteropathogenic bacteria, coronaviruses and rotaviruses, several *C. perfringens* factors can contribute to initial epithelial damage, such as collagenase (kappa toxin), hyaluronidase (mu toxin) and mucinase [38–41]. More research is needed to investigate the role of these factors in the pathogenesis of necrohemorrhagic enteritis and the protective effect of neutralizing antibodies against these proteins.

In this study, we used the calf intestinal loop model to evaluate the vaccine potential of *C. perfringens* alpha toxin. Ideally, vaccinated animals should be challenged with crude toxins or bacterial cultures to obtain conclusive evidence that vaccination against *C. perfringens* alpha toxin protects against bovine necrohemorrhagic enteritis. However, no challenge model for testing vaccine candidates in calves is yet available [19, 31, 42]. The intestinal loop model remains currently the best available model.

In conclusion, this study shows that the non-toxic C-terminal domain of alpha toxin is a promising antigen for vaccine development. Although antibodies against *C. perfringens* alpha toxin neutralize alpha toxin activity and *C. perfringens*-induced endothelial cytotoxicity in vitro, antibodies against alpha toxin alone are inadequate for complete neutralization of *C. perfringens*-induced necrosis in the intestinal loop model of bovine necrohemorrhagic enteritis. The development of a multivalent vaccine combining the C-terminal fragment of alpha toxin with other still unidentified *C. perfringens* virulence factors might be necessary for complete protection against bovine necrohemorrhagic enteritis.
